# Interaction effect of comorbid depression and proactive positivity coping strategy on the 1-year survival of patients with advanced cancer: a nationwide multicentre study in South Korea

**DOI:** 10.1186/s12888-025-06972-4

**Published:** 2025-06-02

**Authors:** Ju Youn Jung, Je-Yeon Yun, Jung Hun Kang, Su-Jin Koh, Yu Jung Kim, Seyoung Seo, Jung Hoon Kim, JaeKyung Cheon, Eun Joo Kang, Eun-Kee Song, Eun Mi Nam, Ho-Suk Oh, Hye Jin Choi, Jung Hye Kwon, Woo-Kyun Bae, Jung Eun Lee, Kyung Hae Jung, EunKyo Kang, Young Ho Yun

**Affiliations:** 1 Seoul National University Medical Research Center, Seoul, Republic of Korea; 2 Hankuk University of Foreign Studies, Seoul, Republic of Korea; 3 Seoul National University Hospital, Seoul, Republic of Korea; 4 Yeongeon Student Support Center, Seoul National University College of Medicine, Seoul, Republic of Korea; 5 Department of Internal Medicine, Gyeongsang National University, Jinju, Republic of Korea; 6 Department of Hematology and Oncology, Ulsan University Hospital, University of Ulsan College of Medicine, Ulsan, Republic of Korea; 7 Department of Internal Medicine, Seoul National University Bundang Hospital, Seoul National University College of Medicine, Seongnam, Republic of Korea; 8 Department of Oncology, Asan Medical Center, University of Ulsan College of Medicine, Seoul, Republic of Korea; 9 Department of Hemato-Oncology, CHA Bundang Medical Center, CHA University, Seongnam, Republic of Korea; 10 Department of Hemato-Oncology, Korea University Guro Hospital, Korea University College of Medicine, Seoul, Republic of Korea; 11 Department of Internal Medicine, Jeonbuk National University Medical School, Jeonju, Republic of Korea; 12 Department of Internal Medicine, Ewha Womans University College of Medicine, Seoul, Republic of Korea; 13 Division of Hemato-oncology, Department of Internal Medicine, GangNeung Asan Hospital, University of Ulsan College of Medicine, Gangneung, Republic of Korea; 14 Division of Medical Oncology, Department of Internal Medicine, Yonsei Cancer Center, Yonsei University College of Medicine, Seoul, Republic of Korea; 15 Department of Internal Medicine, Chungnam National University College of Medicine, Daejeon, South Korea; 16 Division of Hematology-Oncology, Department of Internal Medicine, Hwasun Hospital, Chonnam National University Medical School, Chonnam National University Medical School, Hwasun, Republic of Korea; 17 National Cancer Control Institute, National Cancer Center, Goyang, Republic of Korea; 18 Department of Family Medicine, Seoul National University College of Medicine, Seoul, 03080 Republic of Korea; 19 Department of Human System Medicine, Seoul National University College of Medicine, Seoul, Republic of Korea

**Keywords:** Depression, Coping strategy, Proactive positivity, Advanced cancer, 1-year survival, Cox proportional hazard regression model

## Abstract

**Background:**

Comorbid depression and poor performance status are associated with increased mortality and reduced quality of life in patients with advanced cancer. Coping strategies based on “proactive positivity” may facilitate adaptation to functional decline and limited life expectancy. However, few studies have examined the impact of the interaction between depressive symptoms and coping strategies on survival outcomes in this population. This study investigated the associations of 1-year survival with the interaction between comorbid depression and proactive coping strategies, and performance status, in patients with advanced cancer.

**Methods:**

This was a secondary analysis of data from a multicentre randomized clinical trial of patients with advanced cancer (ClinicalTrials.gov Identifier: NCT03181854). A total of 144 patients who were aware of their cancer diagnosis were recruited from 12 tertiary hospitals across South Korea between October 2017 and October 2018. In this prospective cohort design, participants were stratified into subgroups with higher versus lower levels of baseline proactive coping (proactive positivity) and followed for 1 year to assess survival status. Demographic and socioeconomic data were collected via self-report questionnaires, while cancer diagnosis and treatment information was obtained from attending oncologists. Cancer-related physical functioning, depressive symptoms, and coping strategies were assessed at baseline and at 12 weeks using the Eastern Cooperative Oncology Group Performance Status (ECOG-PS) scale, the Patient Health Questionnaire-9 (PHQ-9), and the Smart Management Strategy for Health Assessment Tool– short form (SAT-SF), respectively. Univariate Cox regression analyses were conducted to identify factors associated with 1-year survival, and a multivariate Cox proportional hazards model was developed to evaluate the predictive impact of performance status, depression, and the interaction between depression and proactive positivity.

**Results:**

In univariate Cox regression models, lower performance status (ECOG-PS = 2; hazard ratio [HR] = 2.33, 95% confidence interval [CI]: 1.25–4.34) and comorbid depression (PHQ-9 ≥ 10; HR = 2.76, 95% CI: 1.72–4.42) were associated with increased risk of not surviving for 1 year. In the multivariate model, among patients with lower proactive positivity (SAT-SF Core strategies score ≤ 66.66/100), comorbid depression was associated with a 363% higher risk of 1-year mortality compared to those without depression (adjusted HR = 4.63, 95% CI: 2.54–8.43). Conversely, the association between depression and 1-year survival was not statistically significant among patients with higher proactive positivity (SAT-SF score > 66.66/100).

**Conclusions:**

Comorbid depression is associated with a significantly higher risk of 1-year mortality in patients with advanced cancer who exhibit lower levels of proactive positivity, but not in those with higher levels of proactive coping. These findings highlight the importance of incorporating assessments of psychological resilience and coping strategies into the clinical management of advanced cancer.

**Trial registration:**

Registry (ClinicalTrials.gov); registration number (NCT03181854); study registration dates [first submitted (2017-06-07), first submitted that met QC criteria (2017-06-07), first posted (2017-06-09)]

## Background

Patients with advanced cancer often experience the distress of a threatened self-identity [1], confusion regarding the meaning of life [2, 3], and fear of death [4], all of which may contribute to or co-occur with depression [5]. To adapt more effectively, individuals with advanced cancer require the capacity to maintain a sense of coherence, perceiving the world and their life as comprehensible, manageable, and meaningful [6]. Although physicians treating adults with advanced cancer increasingly seek to involve patients and their surrogates in advance care planning and end-of-life decision-making, the presence of comorbid anxiety and depression may hinder patients’ engagement in this process [7].

Comorbid depression is highly prevalent among individuals with cancer, affecting over 30% of adults with metastatic disease and their caregivers [8, 9]. In patients with advanced gastric cancer, comorbid depression is associated with more adverse events related to systemic chemotherapy, lower body mass index, and stage IV disease [10]. Among patients with advanced head and neck cancer, the prevalence of depression increases following surgery, including free flap reconstruction, and is associated with factors such as the duration of surgery, length of postoperative hospital stay, time since operation, and speaking difficulties [11]. In response to such distress, patients adopt a range of coping strategies. These include problem-focused efforts aimed at modifying or resolving the stressor, emotion-focused efforts to reduce or regulate distress, and meaning-focused efforts to maintain positive well-being [5].

Coping strategies evolve dynamically across the trajectory of advanced cancer and are frequently influenced by patients’ physical and emotional states, as well as the receipt of difficult prognostic information [5]. Individuals with incurable advanced cancer often strive to reduce the emotional pain linked to their impending death while preserving meaningful connections to life. They do so by employing coping strategies rooted in togetherness, engagement, hope, and continuity [12]. When introducing new coping strategies that align with a patient’s functional status, healthcare teams have found utility in behavioural strategies such as problem-solving and seeking social support, emotional strategies such as maintaining hope and contemplating future possibilities, and existential strategies involving meaning-making [13]. Long-term cancer survivors can also serve as valuable resources, sharing experiences of suicidal ideation and mental health challenges while offering support within survivor communities [14]. Moreover, hope has been identified as a predictor of survival in advanced cancer [15]. To enhance coping and sustain hope among patients with limited life expectancy, healthcare teams should emphasize achievable strategies, including symptom control, emotional support, dignity preservation, and realistic day-to-day goal setting [16].

Regarding the relationship between comorbid depression and coping strategies in patients with advanced cancer, existing evidence demonstrates interactions among depressive symptoms, coping mechanisms, and quality of life (QOL). First, depressive and anxiety symptoms have been shown to mediate the positive association between positive reframing as a coping strategy and improved QOL and emotional well-being in patients with advanced lung cancer [17]. Second, both social support and spiritual coping mediate the relationship between hope and depression in individuals with advanced cancer [18]. Third, among terminally ill patients with prognostic awareness, greater use of positive reframing is associated with better QOL and fewer depressive symptoms [19]. Fourth, increased use of approach-oriented coping and reduced reliance on avoidant coping are linked to higher QOL and lower levels of depression in patients with incurable advanced cancer [20].

Despite these findings, limited information exists regarding the impact of the interaction between depressive symptoms and coping strategies on survival in patients with advanced cancer. Therefore, the present study examined the potential associations among 1-year survival, a baseline coping strategy characterized by “proactive positivity,” and comorbid depression in patients with advanced cancer. Utilizing data from a multicentre randomized clinical trial evaluating early palliative care in advanced cancer patients, i.e. those with either stage IV disease at initial diagnosis or with recurrent cancer following prior treatment [Clinical Trial Number: NCT03181854], we conducted a secondary analysis using a prospective cohort design. We reclassified participants into subgroups with higher versus lower levels of baseline proactive positivity and followed them for 1 year to track survival duration and determine 1-year survival status (survived or deceased). Given the well-established association between performance status and survival length in advanced cancer [21–24], we included both the interaction between coping strategy and depression, as well as baseline physical functioning, as potential predictive variables in a multivariate Cox proportional hazards regression model of 1-year survival. We hypothesized that comorbid depression would be associated with increased odds of not surviving for 1 year. Furthermore, we hypothesized that the impact of comorbid depression on 1-year survival might differ depending on the level of engagement in proactive coping strategies among patients with advanced cancer.

## Methods

### Study participants

In the present study, we conducted a secondary analysis of data from a multi-centre randomized clinical trial involving patients with advanced cancer [Clinical Trial Registry (ClinicalTrials.gov); Clinical Trial Number: NCT03181854]. Some results from this trial, particularly those concerning the effect of earlier palliative care interventions on the quality of life of patients with advanced cancer, have been published previously [25].

The primary aim of the original randomized controlled trial was to test the superiority of an earlier palliative care intervention, consisting of outpatient consultations with a palliative care physician every 3 weeks and telephone coaching every 2–3 weeks during the first 6 months, compared to a control condition that involved usual palliative care provided upon request over 12 months. During the second 6 months, patients in the intervention group could also receive usual palliative care upon request.

Study participant recruitment and enrolment were conducted between October 2017 and October 2018 (accrual period: 12 months). Participants were randomized in a 1:1 ratio to either the intervention or control arm. The baseline assessment and scheduled follow-ups (at weeks 12, 18, and 24, at 1 year, and post-mortem) concluded in June 2019 (maximum follow-up duration: 20 months).

The estimated median survival time was hypothesized as 6 months for the control group, reflecting survival data for stage IV advanced cancer patients aged approximately 60 years, with an average 1.5-year history of hepatobiliary or pancreatic cancer (which accounted for 50% [*n* = 73/144] of the study participants). Approximately 50 out of 100 people with this status are expected to survive for 2–10 months [26]. The median survival time in the intervention group was hypothesized to be 12.5 months, assuming a potential gain in life expectancy of approximately 0.5 months in patients with a baseline life expectancy of ≤ 12 months.

To achieve a power of 0.8 (β = 0.2) and a significance level (α) of 0.05, the required sample size was calculated as 134 participants [27]. Accounting for an expected dropout rate of approximately 7% (~ 10 participants), the final sample size was set at 144 participants.

Eligible participants were recruited from 12 tertiary hospitals across the Republic of Korea between October 2017 and October 2018. Four of the participating hospitals were ranked among the “World’s Best Specialized Hospitals, 2025” in oncology: Asan Medical Centre (3rd), Seoul National University Hospital (8th), Seoul National University Bundang Hospital (57th), and Chonnam National University Hwasun Hospital (116th) (https://r.statistita.com/en/healthcare/worlds-best-specialized-hospitals-2025/ranking/).

The inclusion criteria were as follows: age ≥ 20 years; histologically or cytologically confirmed advanced cancer of a solid tumour; Eastern Cooperative Oncology Group performance status (ECOG-PS) score of 0 (fully active), 1 (ambulatory and capable of light or sedentary work), or 2 (ambulatory and capable of self-care but unable to perform work activities; “up and about” > 50% of waking hours); and an estimated life expectancy of ≤ 12 months, as determined by the attending oncologist. All participating oncologists were active members of the Korean Society of Medical Oncology (KSMO; http://eng.ksmo.or.kr/main.html) and had comparable clinical expertise.

The exclusion criteria were an inability to speak, understand, or write in Korean; medical conditions that would hinder compliance with the clinical trial protocol (e.g. dyspnoea), as determined by the referring physician; suspension of all cancer treatments; and prior or ongoing palliative care consultation.

This study was approved by the Institutional Review Board (IRB) of Seoul National University College of Medicine and Hospital (Seoul, Republic of Korea; IRB No. 1602-143-745) and was conducted in accordance with the ethical standards of the 1975 Declaration of Helsinki and its 2013 amendments. Written informed consent was obtained from all participants.

### Measures: demographic, socio-economic, and cancer-related clinical information

At baseline, demographic (age, sex, marital status, and religious practice) and socio-economic (educational attainment, monthly household income, and residential area) information was collected via self-reported questionnaires. Clinical information related to cancer diagnosis at baseline (primary tumour site, number of metastatic sites, and timing of classification as advanced cancer—stage IV at initial diagnosis vs. recurrence after prior treatment) and treatment received at the 12-week follow-up (standard chemotherapy, participation in clinical trials, or outpatient palliative care) was obtained from each patient’s attending oncologist using self-administered questionnaires.

Participants also self-reported their cancer-related physical functioning using the ECOG-PS scale [28] at both baseline and the 12-week follow-up. The ECOG-PS categorizes performance status into six levels:

- 0: Fully active, able to carry out all pre-disease activities without restriction.
- 1: Restricted in physically strenuous activity but ambulatory and able to carry out light or sedentary work.
- 2: Ambulatory and capable of all self-care but unable to carry out any work activities; “up and about” > 50% of waking hours.
- 3: Capable of only limited self-care; confined to bed or chair for > 50% of waking hours.
- 4: Completely disabled; unable to perform self-care and totally confined to bed or chair.
- 5: Deceased.

### Measures: comorbid depression

Depressive symptoms were assessed using the Patient Health Questionnaire-9 (PHQ-9), a nine-item self-report measure [29, 30], administered at both baseline and the 12-week follow-up. Each item was rated on a four-point Likert scale. Total scores were categorized as follows: none–minimal (0–4), mild (5–9), moderate (10–14), moderately severe (15–19), and severe (20–27) [29]. Participants with a PHQ-9 score ≥ 10 at baseline were classified as having depression; those with scores < 10 were considered not to have depression [31].

### Measures: use of coping strategies

Coping strategies were assessed at baseline and at the 12-week follow-up using the Smart Management Strategy for Health Assessment Tool– short form (SAT-SF), a validated self-report questionnaire [32, 33]. The SAT-SF evaluates core strategies (SAT-SF Core; 10 items measuring proactive positivity), preparation strategies (SAT-SF Preparation; 10 items on goal-oriented behaviour), and implementation strategies (SAT-SF Implementation; 10 items assessing self-regulation) [33].

Each item is rated on a 4-point Likert scale (1 = Not at all true, 2 = A little true, 3 = Mostly true, 4 = Very true). Sub-scores are standardized to a 0–100 scale using a validated scoring algorithm (https://qol.eortc.org/manual/scoring-manual/), with higher scores indicating more effective coping. Based on previous validation studies [32–34], participants were categorized into two subgroups for the SAT-SF Core domain: higher coping strategy use (> 66.66) and lower coping strategy use (≤ 66.66).

### Measures: patient survival at 1-year follow-up

Survival status was monitored for 1 year from the time of enrolment using data collected from participants, family members, and physicians. Follow-up assessments occurred at 12, 18, and 24 weeks, and again at 12 months, in either inpatient or outpatient settings. For patients lost to follow-up, survival status was verified through their attending physician. Patients for whom no contact could be established were classified as “unable to contact.”

### Statistical analysis

This study examined the potential associations among 1-year survival, baseline use of proactive positive coping strategies, and comorbid depression in patients with advanced cancer. Accordingly, participants were reclassified into two groups based on their baseline SAT-SF Core strategies scores, as supported by previous validation studies [32–34]: a higher proactive positivity group (SAT-SF Core score > 66.66) and a lower proactive positivity group (SAT-SF Core score ≤ 66.66). Participants were followed over a 1-year period to assess survival outcomes (survived vs. deceased).

First, descriptive analyses were conducted to assess the distribution and between-group differences in demographic, socio-economic, and clinical characteristics. Between-group comparisons were conducted using independent t-tests for continuous variables (age, number of metastatic sites, and SAT-SF sub-scores) and chi-squared tests for categorical variables, including sex; educational attainment; monthly household income (< USD 3,000 vs. ≥ USD 3,000); residential area (rural/suburban vs. metropolitan); religious practice (yes vs. no); primary tumour site; reason for advanced cancer classification (stage IV at initial diagnosis vs. recurrence after prior treatment); type of cancer treatment at the 12-week time point (standard chemotherapy vs. clinical trial participation vs. outpatient palliative care); ECOG-PS; comorbid depression based on PHQ-9 severity category (none [0–4], mild [5–9], moderate [10–14], moderately severe [15–19], and severe [20–27]); and 1-year survival outcome.

Additionally, within each group (higher vs. lower proactive positivity at baseline), paired t-tests were used to examine within-group changes in depressive symptom severity (PHQ-9 scores), SAT-SF sub-scores, and ECOG-PS between baseline and the 12-week follow-up. A *p*-value of < 0.05 was considered statistically significant in these comparisons.

Second, univariate Cox proportional hazards regression analyses were performed to identify factors associated with 1-year survival, with a significance threshold set at *p* < 0.01. Third, a multivariate Cox proportional hazards regression model was constructed to assess the interaction between baseline comorbid depression and baseline proactive positivity as a predictor of 1-year survival. This model also included all covariates that were statistically significant in the univariate analyses (*p* < 0.05).

Kaplan–Meier survival curves of the interaction between proactive positivity and depression were generated using the MatSurv function [35] in MATLAB software (version R2022a; https://www.mathworks.com). Cox proportional hazards model fitting, along with the estimation of hazard ratios (HRs) and 95% confidence intervals (CIs), was performed using the “*coxph*” and “*Surv*” functions of the R package survival (https://cran.r-project.org/web/packages/survival/index.html).

## Results

### Characteristics of study participants

A total of 144 patients with advanced cancer were enrolled in this study (Table 1). The sample included 83 males (57.6%) and 61 females (42.4%), with a mean age of 60.7 years (SD = 7.2). At baseline, the prevalence of higher coping strategy use was as follows: proactive positivity (SAT-SF Core strategies score > 66.66), 36.8% (*n* = 53/144); strategic pursuit of purpose (SAT-SF Preparation strategy score > 66.66), 18.1% (*n* = 26/144); and sustainable self-governance (SAT-SF Implementation strategy score > 66.66), 11.8% (*n* = 17/144).

**Table 1:** Sociodemographic and clinical characteristics of advanced cancer patients with higher versus lower baseline proactive positivity

Variables		Category	Higher proactive positivity at baseline (SAT-SF Core strategies > 66.66; *N* = 53)	Lower proactive positivity at baseline (SAT-SF Core strategies ≤ 66.66; *N* = 91)	*P* value
Age (years)		Mean (SD)	60.1 (8.6)	60.9 (8.9)	0.570
Sex		Male/Female	34/19	49/42	0.228
Educational achievement		< High school/≥ High school	22/31	46/45	0.295
Monthly household income		< 3,000 USD/≥ 3,000 USD	42/11	87/4	0.002*
Residential area		Rural or suburban/Metropolitan area	37/16	49/42	0.060
Marital status		Unmarried/Married	8/45	27/64	0.049
Religious practice		No/Yes	24/29	37/54	0.588
Primary tumour site		Hepato-pancreato-biliary/stomach-duodenum-colon/lung/breast/urinary-genital/thymoma-sarcoma	28/11/9/2/2/1	45/15/9/15/6/1	0.223
Number of metastatic sites		Mean (SD)	1.7 (0.8)	2.0 (1.2)	0.063
Time-point of diagnosis with advanced cancer		Stage 4 at initial diagnosis/Recurrence after treatment	33/20	58/33	0.860
ECOG-PS	Baseline	0 (Fully active)/1 (remains ambulatory and able to carry out work of a light or sedentary nature)/2 (ambulatory and capable of all self-care, but unable to carry out any work activities; “up and about” > 50% of waking hours)/3 (capable of only limited self-care; confined to bed or chair > 50% of waking hours)/4 (completely disabled; totally confined to bed or chair)	1/49/3/0/0	6/68/17/0/0	0.032
	12-week follow-up *n* = 96/118 (alive)]		0/31/4/2/1	3/47/3/3/2	0.572
	Baseline vs. 12 wk *(P values)*		0.023	0.055	
Depression (PHQ-9 total score)	Baseline [*N* = 143]	No (0–4)/mild (5–9)/Moderate (10–14)/moderately severe (15–19)/severe (20–27)	22/17/10/3/1	34/26/21/6/4	0.876
	12-week follow-up [*n* = 80/118 (alive)]		11/10/8/1/1	30/12/6/1/0	0.161
	Baseline vs. 12 wk *(P values)*		0.073	0.204	
SAT-SF strategies	Baseline	Core strategies (Proactive positivity)	83.52 (12.08)	37.08 (19.13)	< 0.001*
		Preparation strategies (Strategic pursuit of purpose)	62.96 (22.30)	28.32 (19.35)	< 0.001*
		Implementation strategies (Sustainable self-governance)	57.26 (20.41)	27.95 (17.14)	< 0.001*
	12-week follow-up [*n* = 78/118 (alive)]	Core strategies (proactive positivity)	68.22 (21.32)	40.89 (24.61)	< 0.001*
		Preparation strategies (Strategic pursuit of purpose)	55.33 (21.97)	30.79 (24.81)	< 0.001*
		Implementation strategies (Sustainable self-governance)	53.66 (22.22)	34.35 (23.10)	< 0.001*
	Baseline vs. 12 wk *(P values)*	Core/Preparation/Implementation	< 0.001*/0.038/0.239	0.190/0.586/0.032	
1-year survival		Survived/Not survived/censored	17/29/7	40/46/5	0.158
Type of cancer treatment from baseline to 12-wk follow-up	Variable	Category [*n* = 95; 92/118 (alive) + 3/26 (expired) on 12th week]	Standard chemotherapy (Y/N)	Advanced cancer treatment (clinical trial) (Y/N)	Outpatient palliative care (Y/N)
	Proactive positivity (SAT-SF Core strategies): baseline	Higher proactive positivity at baseline (> 66.66)	35/3	1/34	12/26
		Lower proactive positivity at baseline (≤ 66.66)	51/6	2/49	11/46
		Higher vs. Lower *(P values)*	0.668	0.070	0.171
	Depression (PHQ-9 total score): baseline	No depression (0–9)	81/8	3/78	21/68
		Depression (10–27)	5/1	0/5	2/4
		No depression vs. Depression (*P values)*	0.534	0.661	0.590

*P < 0.01; ECOG-PS, Eastern Cooperative Oncology Group performance status; USD, United States dollar; PHQ-9, Patient Health Questionnaire-9; SAT, Smart management strategies for health Assessment Tool

Patients in the higher proactive positivity group (*n* = 53) had a significantly higher monthly household income (≥ USD 3,000) compared to those in the lower proactive positivity group (*n* = 91) (*p* = 0.002). The prevalence of comorbid depression, defined as a PHQ-9 total score ≥ 10, was 31.3% (*n* = 45/144) at baseline and did not significantly differ between the higher and lower proactive positivity groups (*p* > 0.05).

Among patients in the higher proactive positivity group who survived the first 12 weeks (*n* = 43), the mean SAT-SF Core strategies score significantly declined from 83.52 (baseline) to 68.22 at the 12-week follow-up (*p* < 0.001). There were no statistically significant differences in the types of cancer treatment received between subgroups with vs. without comorbid depression or between subgroups with higher vs. lower proactive positivity at baseline (all *p* > 0.05; Table 1).

### Cox regression models of 1-year survival in patients with advanced cancer

Univariate Cox regression analyses (Table 2) revealed that 1-year mortality was significantly associated with impaired physical performance (ECOG-PS score = 2 vs. 0–1; HR = 2.33; 95% CI: 1.25–4.34) and comorbid depression (PHQ-9 score ≥ 10 vs. < 10; HR = 2.76; 95% CI: 1.72–4.42; *p* < 0.001).

**Table 2:** Univariate Cox regression analyses: 1-year survival, sociodemographic factors, and clinical characteristics at baseline

Variables	Category	Crude HR (95% CI)	*P* value
Age	≥ 65 years	1	0.97
	< 65 years	1.01 (0.62–1.65)	
Sex	Female	1	0.07
	Male	1.54 (0.96–2.45)	
Educational achievement	≥High school	1	0.65
	< High school	1.11 (0.71–1.75)	
Monthly household income	≥ 3,000 USD	1	0.49
	< 3,000 USD	1.22 (0.69–2.15)	
Residential area	Metropolitan area	1	0.10
	Rural or suburban	1.51 (0.93–2.45)	
Marital status	Unmarried	1	0.043
	Married	0.60 (0.36–0.99)	
Religious practice	Yes	1	0.06
	No	0.62 (0.38–1.02)	
Primary tumor site	Lung / breast / urinary-genital / thymoma-sarcoma	1	0.746
	Stomach-duodenal-colon / hepato-biliary-pancreatic	0.92 (0.56–1.52)	
Number of metastatic sites	3 sites >	1	0.251
	3 sites ≤	1.36 (0.81–2.29)	
Reason for diagnosis of advanced cancer	Stage 4 at initial diagnosis	1	0.26
	Recurrence after treatment	0.76 (0.47–1.23)	
Early palliative care	Consultation with palliative care physician & telephone coaching	1	0.289
	Usual palliative care provided if desired	0.78 (0.49–1.23)	
**Performance status (ECOG-PS): baseline**	0 (Fully active)/ 1 (remains ambulatory and able to carry out work of a light or sedentary nature)	1	**0.008**
	2 (ambulatory and capable of all self-care, but unable to carry out any work activities; “up and about” > 50% of waking hours)	**2.33 (1.25–4.34)**	
**Depression (PHQ-9 total score): baseline**	No (0–4) / mild (5–9)	**1**	**< 0.001**
	Moderate (10–14) / moderately severe (15–19) / severe (20–27)	**2.76 (1.72–4.42)**	

*P < 0.01. HR, hazard ratio; CI, confidence interval; ECOG-PS, Eastern Cooperative Oncology Group performance status; USD, United States dollar; PHQ-9, Patient Health Questionnaire-9; SAT, Smart management strategies for health Assessment Tool

**Fig. 1:**
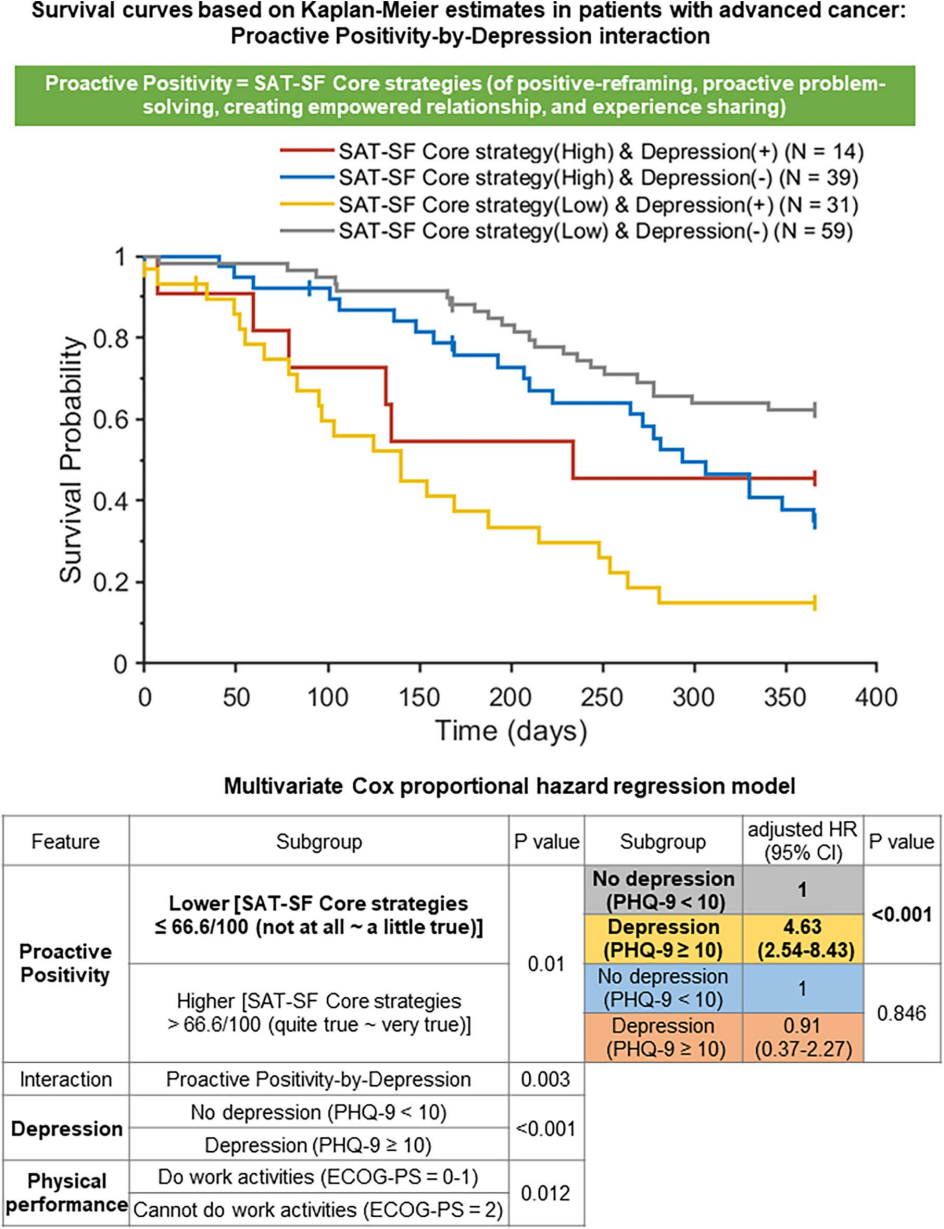
Interaction effect of proactive positivity (Core strategies SAT-SF sub-score) and depression (total score on the PHQ-9) on the 1-year survival probability of patients with advanced cancer. Comorbid depression was associated with higher odds of not surviving for 1 year in advanced cancer patients with lower proactive positivity, but not in those with higher proactive positivity. Upper panel shows Kaplan–Meier survival probability plots. Lower panel displays the adjusted HR of not surviving for 1 year, calculated through multivariate Cox proportional hazards regression. CI, confidence interval; ECOG-PS, Eastern Cooperative Oncology Group performance status; HR, hazard ratio; PHQ-9, Patient Health Questionnaire-9; SAT-SF, Smart Management Strategy for Health Assessment Tool– short form

In the multivariate Cox proportional hazards model including physical performance, depression, proactive positivity, and the interaction term between proactive positivity and depression, several predictors remained significantly associated with 1-year survival (Fig. 1). These included physical performance (ECOG-PS; *p* = 0.012), comorbid depression (PHQ-9; *p* < 0.001), proactive positivity (SAT-SF Core strategies; *p* = 0.010), and the interaction between proactive positivity and depression (*p* = 0.003).

Among patients with lower proactive positivity at baseline (SAT-SF Core score ≤ 66.66), comorbid depression was associated with a markedly increased risk of 1-year mortality. These individuals had a 363% higher risk of not surviving the 1-year follow-up compared to their non-depressed counterparts (adjusted HR [aHR] = 4.63; 95% CI: 2.54–8.43; *p* < 0.001). In contrast, among patients with higher proactive positivity (SAT-SF Core score > 66.66), comorbid depression was not significantly associated with 1-year mortality (*p* = 0.846).

## Discussion

### Lower proactive positivity in advanced cancer patients with comorbid depression is associated with higher risk of not surviving 1 for year

In the univariate Cox proportional hazards regression model, comorbid depression at baseline (PHQ-9 total score ≥ 10) was associated with significantly increased odds of not surviving for 1 year (aHR = 2.76; Table 2). This association was particularly pronounced in the subgroup of patients with lower use of the proactive positivity coping strategy at baseline (aHR = 4.63; Fig. 1). These findings aligned with previous studies demonstrating that comorbid depression is associated with increased mortality, reduced QOL, and challenges in life planning among patients with advanced cancer.

First, a prospective cohort study using data from the U.S. National Health and Nutrition Examination Survey found that lower PHQ-9 scores (0–4) were associated with reduced risks of all-cause and non-cancer mortality compared to higher scores (≥ 10) [36]. Second, in the large population-based PROFILES study, which included various cancer types, motivational anhedonia was significantly associated with increased mortality over time, even after adjusting for clinical and sociodemographic variables [37]. Similarly, sentiment analysis of social media posts revealed that short-term cancer survivors exhibited significantly more depression-related content and anxiety-laden language than long-term survivors [14].

Third, in patients with gynaecologic cancers, 2-year disease-free survival rates were significantly lower in those with comorbid depression than in those without depression [38]. Patients with ≥ stage 3 cancer, a history of at least five chemotherapy regimens, post-chemotherapy side effects, and comorbid depression were also at greater risk of cancer progression [38]. Fourth, both progression-free and overall survival within 3 years of systemic chemotherapy were significantly poorer among advanced gastric cancer patients with comorbid depression and anxiety compared to those with normal emotional states [10]. Lastly, among patients with non-small cell lung cancer, the quality-adjusted life expectancy was found to be shorter than the disability-free life expectancy due to the burden of discomfort and comorbid depression [39].

### Comorbid depression loses predictive power in patients with higher proactive positivity

In contrast, among patients with advanced cancer who exhibited higher levels of proactive positivity, characterized by proactive problem-solving, positive reframing, the creation of empowered relationships, and sharing of experiences, the predictive impact of comorbid depression on 1-year survival was no longer statistically significant. These findings were consistent with previous studies on patients with advanced cancer lacking curative treatment options, in which coping strategies such as confrontational coping, social support seeking, planned problem-solving, and positive reappraisal were negatively correlated with hopelessness and depressive symptoms [40].

Several mechanisms may explain how proactive coping contributes to improved survival and psychological outcomes. First, unresolved daily life problems can significantly impair the quality of care, especially at the end of life [41]. Second, the strategy of positive reframing may serve to foster hope in a manner congruent with the clinical realities of advanced cancer. Hope is conceptualized as a future-oriented expectancy comprising goals [42, 43], pathways (the perception that strategies or routes to achieve goals are available) [44, 45], and agency (belief in one’s capacity to pursue goals despite obstacles) [43]. For some patients, even participation in clinical trials may function as a pathway that allows them to contribute to science and benefit future patients [46]. Conversely, patients with lower levels of hope often report feelings of helplessness, fatalistic acceptance, and anxious preoccupation with their illness [47]. Indeed, hopelessness has been shown to predict mortality over a 4-year period in patients with advanced cancer [48].

Third, utilization of social support from family, friends, or healthcare providers may provide a psychologically safe environment in which patients can process their negative experiences [49], thereby reinforcing and expanding sources of hope [15]. In contrast, limited social support is associated with lower perceived dignity in patients with advanced cancer [50], as well as reduced overall survival, particularly in those with advanced gastrointestinal cancers [51].

### Limitations

This study had several limitations. First, we did not examine the directionality of the interactions between comorbid depression and proactive coping strategies in patients with advanced cancer. Future studies using phenotype network approaches [52, 53] may provide more nuanced insights into directional, item-level associations among depressive symptoms, proactive coping strategies, cancer-related daily physical functioning, and 1-year survival. Second, the participants in this study were exposed to varying interventions during the initial 3 months: either usual care (with palliative care available upon request) or telephone-based coaching combined with consultations from a palliative care team. However, by the 12-week time point, patterns of cancer treatment, including standard chemotherapy, participation in clinical trials involving anti-cancer agents, and outpatient palliative care, were similar between subgroups with higher and lower proactive coping levels (Table 2), suggesting minimal confounding due to treatment differences. Third, proactive coping strategies were assessed using self-reported questionnaires without accompanying behavioural data. Future research should include behavioural analyses of real-world daily activities to determine the extent to which self-reported coping strategies align with actual behaviour [54, 55].

## Conclusions

Comorbid depression is associated with a significantly higher risk of not surviving for 1 year in advanced cancer patients who demonstrate lower proactive positivity. However, this association was not observed in patients exhibiting higher levels of proactive positivity. These findings demonstrated the importance of integrating psychological assessments into the care of patients with advanced cancer. Specifically, treatment plans should be tailored to address comorbid depression in conjunction with assessments of proactive positivity and functional performance status, particularly in patients unable to carry out any work-related activities, as these factors may predict reduced 1-year survival.

## Data Availability

The datasets used and/or analysed during the current study are available from the corresponding author (lawyun08@gmail.com) on reasonable request.
